# Meeting Report: Hazard Assessment for Nanoparticles—Report from an Interdisciplinary Workshop

**DOI:** 10.1289/ehp.10327

**Published:** 2007-08-14

**Authors:** John M. Balbus, Andrew D. Maynard, Vicki L. Colvin, Vincent Castranova, George P. Daston, Richard A. Denison, Kevin L. Dreher, Peter L. Goering, Alan M. Goldberg, Kristen M. Kulinowski, Nancy A. Monteiro-Riviere, Günter Oberdörster, Gilbert S. Omenn, Kent E. Pinkerton, Kenneth S. Ramos, Kathleen M. Rest, Jennifer B. Sass, Ellen K. Silbergeld, Brian A. Wong

**Affiliations:** 1 Environmental Defense, Washington, DC, USA; 2 Woodrow Wilson International Center for Scholars, Washington, DC, USA; 3 Rice University, Houston, Texas, USA; 4 National Institute for Occupational Safety and Health, Morgantown, West Virginia, USA; 5 Procter and Gamble, Cincinnati, Ohio, USA; 6 U.S. Environmental Protection Agency, Research Triangle Park, North Carolina, USA; 7 Food and Drug Administration, Rockville, Maryland, USA; 8 Johns Hopkins Bloomberg School of Public Health, Baltimore, Maryland, USA; 9 North Carolina State University, Raleigh, North Carolina, USA; 10 University of Rochester, Rochester, New York, USA; 11 University of Michigan, Ann Arbor, Michigan, USA; 12 University of California at Davis, Davis, California, USA; 13 University of Louisville, Louisville, Kentucky, USA; 14 Union of Concerned Scientists, Cambridge, Massachusetts, USA; 15 Natural Resources Defense Council, Washington, DC, USA; 16 The Hamner Institutes for Health Sciences, Research Triangle Park, North Carolina, USA

**Keywords:** nanomaterials, nanoparticle, nanotechnology, nanotoxicology, particle toxicology

## Abstract

In this report we present the findings from a nanotoxicology workshop held 6–7 April 2006 at the Woodrow Wilson International Center for Scholars in Washington, DC. Over 2 days, 26 scientists from government, academia, industry, and nonprofit organizations addressed two specific questions: what information is needed to understand the human health impact of engineered nanoparticles and how is this information best obtained? To assess hazards of nanoparticles in the near-term, most participants noted the need to use existing *in vivo* toxicologic tests because of their greater familiarity and interpretability. For all types of toxicology tests, the best measures of nanoparticle dose need to be determined. Most participants agreed that a standard set of nanoparticles should be validated by laboratories worldwide and made available for benchmarking tests of other newly created nanoparticles. The group concluded that a battery of tests should be developed to uncover particularly hazardous properties. Given the large number of diverse materials, most participants favored a tiered approach. Over the long term, research aimed at developing a mechanistic understanding of the numerous characteristics that influence nanoparticle toxicity was deemed essential. Predicting the potential toxicity of emerging nanoparticles will require hypothesis-driven research that elucidates how physicochemical parameters influence toxic effects on biological systems. Research needs should be determined in the context of the current availability of testing methods for nanoscale particles. Finally, the group identified general policy and strategic opportunities to accelerate the development and implementation of testing protocols and ensure that the information generated is translated effectively for all stakeholders.

Close to 400 manufacturer-identified nanotechnology-based consumer products are now on the market (Woodrow Wilson International Center for Scholars 2007). Using increasingly sophisticated levels of control over the assembly of atoms and molecules to form substances and devices, nanotech companies are exploiting the size-dependent properties of nanostructured materials for applications ranging from cosmetics to fuel cells (Colvin 2003). Yet our understanding of the potential toxicity of nanoparticles remains rudimentary (Colvin 2003; Oberdörster et al. 2005b). To determine whether the unique chemical and physical properties of new nanoparticles result in specific toxicologic properties, the nanotechnology community needs new ways of evaluating hazard and ultimately assessing risk (Nel et al. 2006). These new strategies must also consider the complexities inherent to studies of chemical mixtures.

This workshop’s assessment of the novel aspects of nanotoxicology built on knowledge gained from prior workshops. In 2004, scientists from many different areas of research came together in Gainesville, Florida, to discuss the emerging field of nanotoxicology (Bucher et al. 2004). They described the challenges facing toxicologists in rigorously characterizing the new materials and in understanding how nanostructures might differentially influence toxicity. This theme was further elaborated in a seminal article by Oberdörster et al. (2005a), which provides a general framework for evaluating the toxicity of engineered nanoparticles.

More detailed questions regarding exactly how to evaluate the potential health impact of engineered nanoparticles remain. This report captures some of the critical information that is still needed to understand the human health impact of engineered nanoparticles and defines mechanisms to begin to acquire this information. Building on the insight from those previous meetings and published articles—that the structure of nanoparticles brings many new challenges to toxicological evaluation—workshop participants were asked to identify both factors that make nanoparticles different and information specific to these differences that is needed to assess nanoparticle hazards. The group was further charged with making recommendations on how to gather and use that additional information to evaluate health hazards associated with these scale-specific properties. Because of the short duration of this workshop, the scope was limited to consideration of toxic properties of nanoparticles. A full evaluation of human health risks will require development of sufficient techniques for assessing exposure to nanoparticles in addition to consideration of toxicity.

E. Silbergeld of Johns Hopkins University opened the workshop with a presentation that explored ways of thinking about and evaluating the potential hazards of nanoparticles. She emphasized focus on the nanoscale interactions that take place in the normal functioning of biological systems in order to understand the positive and negative effects that engineered nanoparticles could have on humans.

For example, because the immune system functions through nanoscale intercellular communications, Lynch et al. (2006) hypothesized that engineered nanoparticles can disrupt these processes with deleterious end results. Specifically, they considered unique interactions between native proteins and the highly curved surfaces of nanoparticles, speculating that the protein shape could be modified after binding. This deformation could expose amino acid residues that are normally buried in the core of the protein, and the immune system would then recognize these newly exposed residues as “cryptic epitopes” and mount an unwanted immune response.

A 2005 study by Zhao et al. (2005) predicted that DNA repair, another vital biological system that operates at the nanoscale, is also susceptible to modification by nanoparticles. Specifically, this study found through computer modeling that the association in water between C_60_ and DNA is stronger than the association between two C_60_ molecules. Therefore, when DNA is damaged, fullerenes can occupy the damaged site, possibly impeding the self-repairing processes of the double-strand DNA and thus negatively impacting the structure, stability, and biological functions of DNA molecules.

These unique interactions between nanoparticles and biological systems afford great promise for medicinal applications, but the unintended consequences could be harmful. We know, for instance, that natural and unintentionally produced ultrafine particulate matter, which is in the same size range as engineered nanoparticles, can carry a broad range of compounds, including polycyclic aromatic hydrocarbons, endotoxin, metals, and other toxic chemicals. These complexes can then damage biological systems (Penn et al. 2005; Schwarze et al. 2006). Gutierrez-Castillo et al. (2006) found that particulate matter with chemicals adsorbed to the surface can damage DNA. These examples suggest that the myriad possible interactions between nanoparticles and harmful environmental chemicals may lead to unique exposures and health risks.

Conventional knowledge about exposure assessment, fate and transport, and current computer models is not necessarily applicable to nanoparticles. But alternative methods such as toxicogenomic technologies, lower-order animal and *in vitro* testing, and ultimately the development of structure activity models could prove useful, providing more rapid testing than traditional animal toxicology tests and allowing for explicit experimental design based on mechanism. The development of alternative methods is an ambitious but necessary goal if the large and growing numbers of nanoparticles are to be adequately assessed for toxicity.

Silbergeld ended her presentation with a charge for the group: to frame its dialogue both to inform the industry on how to look before leaping into the production of new nanoparticles and to provide guidance for those who have already taken that leap.

## Objectives

Participants were asked to address two specific questions:

Question 1: In light of the special physical, chemical, and biological properties of engineered nanoparticles, what information is needed to assess the human health hazards of nanoparticles that are currently in or likely soon to reach commercial production?

Question 2: How can information needed to assess the human health hazards of nanoparticles that are currently in or likely soon to reach commercial production be obtained most expeditiously and efficiently?

The workshop employed alternating plenary and multidisciplinary breakout sessions to consider these questions in detail. Consensus of viewpoints was noted but not required for each of the points considered. Where viewpoints differed has been captured in the discussion.

## Results

### Critical information needs for nanoparticle toxicity

After developing separate lists of critical information needs in the breakout groups, the participants produced the following consensus priority list: *a*) extensive physicochemical characterization; *b*) capacity for macromolecular perturbation; *c*) potential for unintended carriage of toxic molecules; *d*) translocation; *e* ) agglomeration state; *f* ) and chemical composition.

A brief description of each type of information is given below, followed by a discussion of the adequacy of traditional toxicology testing to detect potentially novel forms of toxicity from nanoparticles.

#### Extensive physicochemical characterization

Participants agreed that engineered nanoparticles must be appropriately characterized if the results of toxicity tests are to be interpreted and compared. Characterization would include particle size, size distribution, shape, surface area (some proposed specifying bioavailable surface area), redox potential and properties, purity/identity of contaminants, and catalytic activity, in addition to generation of reactive oxygen species. Materials must be characterized not just initially but repeatedly in order to reflect their physicochemical state in relevant environmental media and their potential transformation at the portal of entry (e.g., the lung, skin, and gastrointestinal tract) and at the target organ. In addition, characterization of the material should be carried out for all phases of the product lifecycle during which exposure or release may be anticipated. Standardized protocols for nanoparticle characterization will be essential to assure consistency across laboratories. Agglomeration state was considered a key physical characteristic of engineered nanoparticles and is discussed separately below.

#### Macromolecular perturbation

Participants agreed on the need to determine the degree to which particular nanoparticles, because of their size and physicochemical properties, could engage in unique interactions with biologically critical macromolecules, including DNA, cytoskeletal elements, collagen, and membrane structures. Participants summarized the evidence assembled to date for such interactions between natural and engineered nanoparticles, including buckyballs with DNA (modeled), carbon nanotubes with cellular nanotubes (hypothetical), and protein adsorption and modification of self-recognition—the so-called cryptic epitopes—discussed above.

#### Carrier role

Nanoparticles are currently the subject of intense pharmacologic research because of their capacity to carry and deliver drugs to specific targets (Chavanpatil et al. 2006). This intrinsic property raises important questions regarding the potential for nanoparticles to carry toxic chemicals that may be present in the environment. Penn et al. (2005) recently described the ability of combustion-generated ultrafine particulate matter to carry toxic chemicals such as polycyclic aromatic hydrocarbons. The association of endotoxin, which can trigger an immune response that complicates the interpretation of toxic response in toxicological tests, is also a concern. Participants agreed on the need to determine the potential for nanoparticles to become carriers for toxic substances, for example, by transporting them within the hollow structures designed for functional payloads (as in nanotubes, dendrimers, and buckyballs) or adsorbed to their surfaces.

#### Translocation

Participants agreed on the need to determine the likelihood that nanoparticles would not be detected by macrophages in the lungs and consequently translocate through alveolar cell membranes into the general circulation. These processes may be critical to determining the ultimate toxicity of nanoparticles. Detailed mechanistic insight into the relationship between nanoparticle composition and physicochemical properties and membrane translocation may reveal ways to “engineer out” the capacity for widespread distribution and subsequent toxicity through modification of surface coatings or other aspects of nanoparticles.

#### Agglomeration state

Nanoparticles can agglomerate rapidly at high concentrations. Agglomeration state has a profound impact on particle size and structure. Individual nanoparticles when agglomerated may create particles that are no longer in the nanoscale size range. Whether those agglomerated particles retain toxic properties of the individual nanoparticles or are capable of subsequently de-agglomerating is a critical question. Agglomeration state as measured in air or water may change inside the body through interactions with biological fluids and proteins and change further still within different tissue/cellular/subcellular compartments that define different optimal affinities for the particles and biological macromolecules.

#### Chemical composition

Some participants pointed out that bulk chemical composition may be a secondary contributor to nanoparticle functionality because interactions with poorly soluble nanoparticles are confined to the surfaces of the material. This means that structural parameters such as surface charge, shape, area, and reactivity are the primary contributors and more immediate mediators of nanoparticle function and toxicity. Particle solubility is another important parameter with respect to the importance of chemical composition. Composition is clearly of greater importance when particles dissolve over time with the body than when they are insoluble and excreted or stored without any appreciable dissolution. In discussions, it was noted that bulk chemical composition may correlate well with biological activity for some materials and should be measured as a matter of course in studies.

#### Adequacy of traditional toxicology tests

For the purposes of this workshop, the term “traditional” refers to those toxicology tests with well-established protocols that have been used in regulatory and risk assessment settings for many years. These are typically but not necessarily whole animal tests employing histopathology for confirmation of toxicity. The term “apical” refers to the fact that the end points assessed in these tests may be the ultimate manifestation of a variety of different pathways of toxicity. Although participants generally agreed that traditional whole animal toxicology tests would likely show end points of toxicity even if the mechanisms were new, some disagreed as to how adequate those apical tests were for detecting all relevant types of toxicity and noted that scientists need to be open to detecting novel end points. Some participants suggested that the use of molecular technologies, including toxicogenomics and proteomics, may be more efficient at identifying novel mechanisms of toxicity.

Participants noted that a number of critical capabilities are currently lacking for nanoparticles, including: *a*) methods to adequately characterize them, especially when present in biological media; *b*) a validated set of *in vitro* assays with which to develop a profile of mechanistic information relevant to nanoparticles (with the possible exception of assays for generation of reactive oxygen species); and *c*) sufficient experience and data to be able to predict the toxicity of nanoparticles on the basis of their physicochemical characteristics or mechanistic assays. In the face of these shortcomings, most of the group agreed on the urgent need both for traditional toxicologic testing on presently available materials, particularly those most likely to have widespread exposures, and for mechanistic assays, to begin assembling the database that will ultimately enable structure–function predictions and acquisition of biological insight into mechanisms of pathogenesis. Many in the group urged caution, however, to ensure that findings of mechanistic assays are not overinterpreted. These themes are elaborated to some degree in the next section.

An additional critical need is the development of absorption–distribution–metabolism and excretion (ADME) methods and studies. The general framework for ADME should be the same for nanoparticles, but the analytical challenges of detecting nanoparticles, as well as their by-products if they are transformed or metabolized, are significant. Some nanoparticles (e.g., quantum dots, magnetic nanocrystals) have special properties that make their real-time *in vivo* imaging straightforward. However, such imaging is not quantitative and must be confirmed by tissue analysis tests. Additionally, heavy reliance on imaging may not capture the dissolution or transformation of particles or their coatings. Nanoparticles that do not have intrinsic fluorescence or other properties that aid visualization present a greater challenge to accurate determination of their ADME characteristics.

### Strategies for developing needed information on nanoparticle toxicity

In reconciling the urgent need for better understanding of nanoparticle toxicity with the fact that many of the methods for obtaining that understanding are in need of development, the participants distinguished two lines of research for nanotoxicology, as noted previously. The first is to develop data on materials to which workers and the general population are already being exposed, and the second is to create new, better methods for accurate and efficient testing of hazards and exposure potential, which will create new knowledge to guide the future development of nanoparticles. In addition to research priorities, participants emphasized the need to provide appropriate worker protection in the absence of data on potential hazards.

The group identified a number of complementary strategies for gathering the information needed to assess nanoparticle toxicity, including:

- promoting cross-disciplinary communication;
- developing a set of “representative nanoparticles” for benchmarking; and
- developing agreement on a tiered testing program.

In addition, participants developed a chart depicting the current availability of test methods for various types of information needed to assess nanoparticle toxicity in order to facilitate research prioritization and methods development.

#### Cross-disciplinary communication

Sufficient information on the risks of nanotechnology will come only through close collaboration among researchers across disciplines. Because in-depth understanding of nanoparticle toxicology requires elucidation of the relationship between physicochemical parameters and mechanisms of toxicity, greater communication is needed between physical chemists skilled in characterization of materials and toxicologists and biologists able to discern mechanisms for toxicity. Participants therefore developed a framework for describing the interrelationships among material chemists, cell biologists, developmental biologists, pharmacokineticists, and toxicologists.

**Figure 1:**
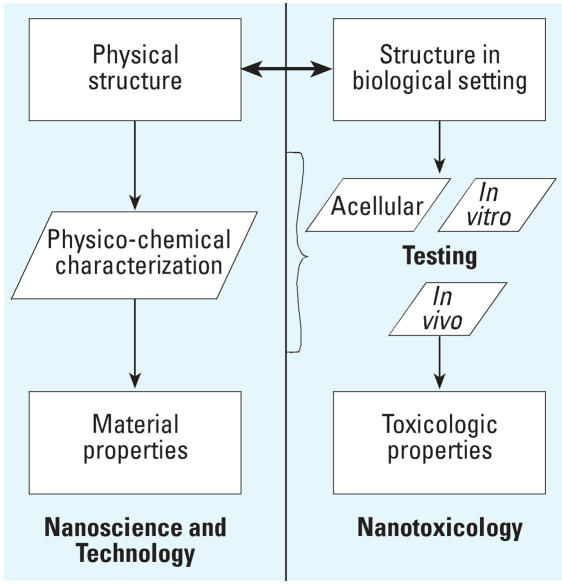
The parallel relationship between material design and material testing (nanotechnology and nanotoxicology).

Nanoparticle design processes (Figure 1, left side) typically seek to correlate physical structure with material properties. This facilitates optimization of technologies and creates a rich framework for scientific study. Nanotoxicology (Figure 1, right side) operates in an analogous context but with a focus on biological and toxicologic properties. Within this framework, information about nanoparticle structure is an important input for biological scientists. Because particle structure (e.g., size, surface coating) in a biological setting can be quite different from that measured in a materials laboratory, an ongoing dialogue between material scientists and biological scientists (dotted two-way arrow) will greatly facilitate the design of safer nanoparticles. In addition, characterization of the physicochemical properties should inform the toxicologic testing process.

Participants also emphasized the need for developing a “community of practice” for nanotoxicology. Elements of this could include the development of standard materials discussed below, as well as workshops and internet listservers to facilitate communication among groups. The development of a standard format for reporting results of nanotoxicology assays, analogous to effort to standardized formatting for toxicogenomics was also suggested [e.g., MIAME (Minimum Information About a Microarray Experiment) 2007 and MIAPE (Minimum Information About a Proteomics Experiment) 2007]. Emphasis for this standardized format would be the novel wealth of information on physicochemical information that will accompany nanotoxicology studies. Last, to promote a broader understanding and shared interpretation of the dynamic scientific literature of nanotoxicology, some participants recommended a standing body modeled on the Cochrane Collaboration, which publishes the Cochrane reviews of evidence for clinical practice. This standing body, for maximum effectiveness and credibility, would comprise representatives of the various stakeholder groups, including academia, industry, government, labor, and nongovernmental organizations.

#### Developing a set of representative nanoparticles for benchmarking

One area of convergent discussion was the need to proceed with full characterization and toxicity testing for a representative set of nanoparticles. The group could not agree on a simple method of selecting a limited set from such a broad and diverse class of materials; even within one class there is currently no way to determine which material is the most representative. The participants’ compromise proposal was to initiate data generation on a group of substances that come from different categories of nanoparticles; ideally, these materials would be closely related to those currently used in commerce. The data to be generated would include full physicochemical characterization, an array of *in vitro* mechanistic tests, and full traditional toxicologic test batteries. Short-term toxicologic testing of nanoparticles (e.g., instillation or aspiration studies with several months of follow-up) was also deemed valuable to facilitate crude benchmarking against well-characterized conventional materials on which these tests have already been performed.

The choice of materials should reflect the priorities of both scientists and regulators, and should be defined within the context of commercial use. Exploratory research, for example, would be best served by using highly pure and monodisperse nanoparticles, whereas the commercially relevant materials of immediate interest to regulators may be more heterogeneous and ill-defined. In addition, testing of these materials should be performed in a manner that reflects the likely routes of exposure based on expected use and lifecycle characteristics of those materials. The group concluded that the selection process will require great care and deliberation beyond the scope of this workshop.

Two further concepts related to representative materials emerged. First, participants agreed that in addition to complete characterization and testing of a small number of materials, it would be useful to run a larger number of materials through some of the same *in vitro* and/or shorter *in vivo* assays to begin interpolating results based on structure or other physicochemical characteristics.

Second, the subset of representative materials being developed and tested should be created in sufficient quantities to be provided to research laboratories as reference material. Given the difficulty of manufacturing nanoparticles and the dynamic nature of the industry, relying on one company to provide a reference material over a period of years was deemed inadvisable. Rather, a small network or consortium should be assembled to produce each reference material to ensure continuity should a business fail or stop production. An essential function of this network would be to establish tests that would ensure that the reference materials were of high quality, and most critically, that they had physicochemical characteristics that were constant over time.

#### Tiered testing program

**Figure 2:**
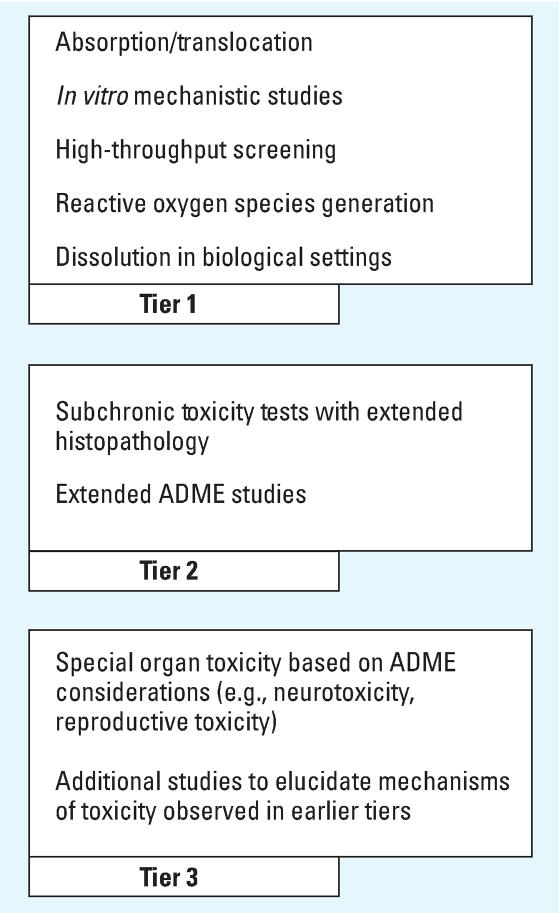
Proposal for a tiered human health hazard assessment program—a decision algorithm for deciding which tier of testing is needed but was not produced during this workshop.

An initial conceptual model for a tiered testing system is presented in Figure 2. Further work is needed to reach consensus on the structure and triggers for such a tiered system. At present, there is insufficient experience with *in vitro* mechanistic studies and other high throughput screening techniques to be able to employ them as initial screens for nanoparticles in place of standard toxicologic tests. Therefore, at present, nanoparticles of concern would need to be considered for tests in the second tier for thorough hazard screening. As greater structure–function and mechanistic understanding develops for nanoparticles, a tiered program such as this should help achieve more efficient hazard screening.

#### Availability of test methods for determining properties of nanoparticles

**Figure 3:**
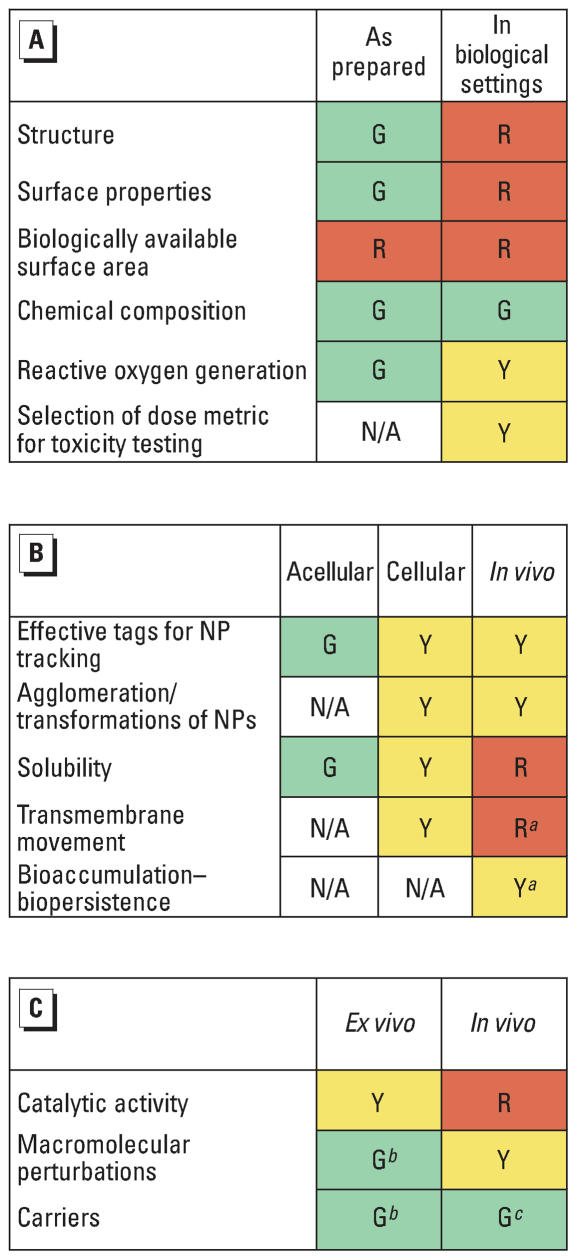
Judged status of available tests for different parameters of dendrimers, metal oxides, and carbon nanotubes. Abbreviations: G, green; N/A, not applicable; NPs, nanoparticles; R, red; Y, yellow. (*A)* Physical chemical characterization; (*B)* ADME/ translocation; and (*C*) biochemistry of nanoparticles. ^***a***^Particles that can be radiolabeled can be more readily assessed for these features. ^***b***^Assays are available but are very slow. ***c***Labeling *in vivo* presents a challenge

To aid in the prioritization of research and test methods development, participants proposed using a rating scheme for the availability of test methods to determine the different types of information relevant to nanoparticle toxicity (Figure 3). A red/yellow/green color coding system was suggested to illustrate the maturity of the available tests. Red indicates there is no agreed upon test method and that the development of one would be a high-priority research need; yellow indicates that test methods are available, but there is no consensus on the proper method; and green indicates that there are tests with sufficiently common usage that they can be considered for standardization within 12–18 months. Green does not indicate that these tests have been validated for regulatory purposes but rather that they are well established for nanoparticles. This table was developed by the participants on the basis of consideration of three types of nanoparticles: dendrimers, metal oxides, and carbon nanotubes. It reflects the best judgment of participants at the time of the meeting and is not intended to necessarily apply to other types of nanoparticles.

Figure 3A addresses information needed for the characterization of physical and chemical properties. Because the physical and chemical properties of nanoparticles can vary depending on whether they are tested as prepared or within biological settings, the availability was considered separately for these different settings. Similarly, Figure 3B considers methods for assessing translocation and ADME properties in acellular, cellular (*in vitro*) and *in vivo* systems, whereas Figure 3C considers methods for assessing special chemistry of nanoparticles both *in vivo* and *ex vivo.*

In addition to considering the availability of methods to assess specific properties of nanoparticles, the participants noted that methods of addressing traditional challenges of whole animal toxicology tests may be especially troublesome for nanoparticles because of the potential for novel mechanisms of toxicity. These challenges include extrapolating between species, including from laboratory animals to man, assessing interindividual variability, and using *in vitro* assays to predict *in vivo* toxicity. Discussing these issues in the same context as the availability of testing methods, participants noted that there are no currently available specific or novel approaches to resolving these challenges for nanoparticles.

## Conclusions

Consistent with previous workshops, most of the attendees concluded that at present, standard toxicologic tests are needed to assess the risks of nanoparticles. Even though opinion diverged on the most relevant tests, there was consensus that, for adequate risk management, nanoparticles nearing commercialization should be subjected to a battery of short-term *in vitro* and *in vivo* tests to determine broadly the effects on key target organs and possible molecular mechanisms of toxicity. The participants recommended eventually developing a tiered testing approach in which primarily *in vitro* screening tests would be designed to uncover particular properties that would then trigger more extensive evaluation. To facilitate the appropriate interpretation of testing results, standard reference materials, testing methods, and reporting formats must be developed for nanoparticles and shared among groups worldwide.

The group further concluded that nanotoxicology research should lead to general principles, ideally predictive in nature, that associate material properties with toxicity. Research toward this goal would require extensive collaboration between nanotechnologists and toxicologists. Because many of the toxicologic tests needed for hazard identification are not well developed for nanoparticles or still require validation, priority should be given to research into tier 1 tests that are not yet easily translated to nanoparticles. And because many of the challenges for nanotoxicology are shared by other specializations within the field of toxicology, further research is needed into the validity of key tests, particularly the predictive nature of *in vitro* for *in vivo* results.

Finally, all participants recognized the importance of effective communication for sharing of information on nanotoxicology and informing science-based public and corporate decision making. One recurrent theme was the opportunity for misunderstanding because of the differences in meaning and perspectives among the various disciplines engaged in the workshop. As this report illustrates, the technical barriers to advancing the science are also significant, and research of the proposed magnitude can only happen with sustained funding from government and industry. To ensure the most effective use of limited research funds, all stakeholders must participate in designing a strong research strategy based on sound science and the consensus of experts worldwide. All parties should also recognize the critical role of public communication in this arena; more than in any other area of nanotechnology, this topic requires strong, neutral, and well-funded public outreach programs that equip consumers and policy-makers to engage in the issues at the highest possible technical level.

## Recommendations

Although some of the following recommendations mirror or build upon previously published recommendations (e.g., Maynard et al. 2006; Oberdörster et al. 2005a), they are also the product of a broader multidisciplinary and multistakeholder dialogue:

- Establish a coordinated federal research agenda in the United States for understanding the human health impacts of nanoparticles—a “Master Research Plan”—with significantly increased funding from all relevant sectors.
- Convene specialized workshops and fund research to advance testing capabilities, including:
  - novel characterization methods for use in biological settings;
  - *in vitro* assay selection and validation for nanoparticle tiered testing;
  - use of physicochemical information to predict and understand biological mechanisms and toxicity;
  - study of ADME issues and their connection to nanomedical research; such information would include where particles distribute over time, tissues and organs in which they may accumulate or deposit, how the body transforms the materials, and when and how they are excreted; and
  - integrative models that provide a complete picture of a nanoparticle’s effects and serve as the basis for predictive correlations for the impacts of novel nanoparticles.
- Develop a multidisciplinary community of practice and information-sharing forum for researchers and all interested stakeholders.
- Develop and make available a set of reference nanoparticles for research laboratories to use as controls/benchmarks in their studies.
- Work toward quantitative risk assessment by selecting a set of three to five specific nanoparticles for full physicochemical characterization, and *in vitro* and *in vivo* testing. Additional work on exposure assessment will also be necessary. This could be accomplished within one country such as the United States or within the context of an intergovernmental group, such as the Organization for Economic Cooperation and Development (OECD).
- Develop a standardized format for reporting the results of nanoparticle toxicological studies, focusing on physicochemical characterization. Engage major scientific journals and government research agencies to require such particle characterization for publication and grant reporting.
- Require that a minimal set of physicochemical information be provided for all commercial products.
- Create a third-party body, similar to the Cochrane model but comprised of stakeholders from all groups, to review and provide authoritative interpretations of research in nanotoxicology.

## References

[b1-ehp0115-001654] Bucher J, Masten S, Moudgil B, Powers K, Roberts S, Walker N (2004). Developing Experimental Approaches for the Evaluation of Toxicological Interactions of Nanoscale Materials. http://www.nanotoxicology.ufl.edu/workshop/images/NanoToxWorkshop.pdf.

[b2-ehp0115-001654] Chavanpatil MD, Khdair A, Panyam J (2006). Nanoparticles for cellular drug delivery: mechanisms and factors influencing delivery. J Nanosci Nanotechnol.

[b3-ehp0115-001654] Colvin VL (2003). The potential environmental impact of engineered nanomaterials. Nat Biotechnol.

[b4-ehp0115-001654] Gutierrez-Castillo ME, Roubicek DA, Cebrian-Garcia ME, De Vizcaya-Ruiz A, Sordo-Cedeno M, Ostrosky-Wegman P (2006). Effect of chemical composition on the induction of DNA damage by urban airborne particulate matter. Environ Mol Mutagen.

[b5-ehp0115-001654] Lynch I, Dawson KA, Linse S (2006). Detecting cryptic epitopes created by nanoparticles. Sci STKE.

[b6-ehp0115-001654] Maynard AD, Aitken RJ, Butz T, Colvin V, Donaldson K, Oberdörster G (2006). Safe handling of nanotechnology. Nature.

[b7-ehp0115-001654] MIAME (Minimum Information About a Microarray Experiment) (2007). Home Page.

[b8-ehp0115-001654] MIAPE (Minimum Information About a Proteomics Experiment) (2007). Home Page.

[b9-ehp0115-001654] Nel A, Xia T, Madler L, Li N (2006). Toxic potential of materials at the nanolevel. Science.

[b10-ehp0115-001654] Oberdörster G, Maynard A, Donaldson K, Castranova V, Fitzpatrick J, Ausman K (2005a). Principles for characterizing the potential human health effects from exposure to nanomaterials: elements of a screening strategy. Part Fibre Toxicol.

[b11-ehp0115-001654] Oberdörster G, Oberdörster E, Oberdörster J (2005b). Nanotoxicology: an emerging discipline evolving from studies of ultrafine particles. Environ Health Perspect.

[b12-ehp0115-001654] Penn A, Murphy G, Barker S, Henk W, Penn L (2005). Combustion-derived ultrafine particles transport organic toxicants to target respiratory cells. Environ Health Perspect.

[b13-ehp0115-001654] Schwarze PE, Ovrevik J, Lag M, Refsnes M, Nafstad P, Hetland RB (2006). Particulate matter properties and health effects: consistency of epidemiological and toxicological studies. Hum Exp Toxicol.

[b14-ehp0115-001654] Woodrow Wilson International Center for Scholars (2007). Nanotechnology Consumer Products Inventory.

[b15-ehp0115-001654] Zhao X, Striolo A, Cummings P (2005). C60 binds to and deforms nucleotides. Biophys J.

